# Dynacortin facilitates polarization of chemotaxing cells

**DOI:** 10.1186/1741-7007-5-53

**Published:** 2007-11-26

**Authors:** Cathryn Kabacoff, Yuan Xiong, Runa Musib, Elizabeth M Reichl, John Kim, Pablo A Iglesias, Douglas N Robinson

**Affiliations:** 1 Department of Cell Biology, Johns Hopkins University School of Medicine, Baltimore, MD 21205, USA; 2 Department of Electrical and Computer Engineering, Johns Hopkins University, Baltimore, MD, USA; 3 Current address: The Henry M. Jackson Foundation for the Advancement of Military Medicine, VPRP/DAIDS/NIAD, 6700-A Rockledge Drive, Room 42A165, Bethesda, MD, USA; 4 Current address: Human Genome Sciences, Inc. 14200 Shady Grove Road, Rockville, MD, USA

## Abstract

**Background:**

Cell shape changes during cytokinesis and chemotaxis require regulation of the actin cytoskeletal network. Dynacortin, an actin cross-linking protein, localizes to the cell cortex and contributes to cortical resistance, thereby helping to define the cell shape changes of cytokinesis. Dynacortin also becomes highly enriched in cortical protrusions, which are sites of new actin assembly.

**Results:**

We studied the effect of dynacortin on cell motility during chemotaxis and on actin dynamics *in vivo *and *in vitro*. Dynacortin enriches with the actin, particularly at the leading edge of chemotaxing cells. Cells devoid of dynacortin do not become as polarized as wild-type control cells but move with similar velocities as wild-type cells. In particular, they send out multiple pseudopods that radiate at a broader distribution of angles relative to the chemoattractant gradient. Wild-type cells typically only send out one pseudopod at a time that does not diverge much from 0° on average relative to the gradient. Though *dynacortin*-deficient cells show normal bulk (whole-cell) actin assembly upon chemoattractant stimulation, dynacortin can promote actin assembly *in vitro*. By fluorescence spectroscopy, co-sedimentation and transmission electron microscopy, dynacortin acts as an actin scaffolder in which it assembles actin monomers into polymers with a stoichiometry of 1 Dyn_2_:1 actin under salt conditions that disfavor polymer assembly.

**Conclusion:**

Dynacortin contributes to cell polarization during chemotaxis. By cross-linking and possibly stabilizing actin polymers, dynacortin also contributes to cortical viscoelasticity, which may be critical for establishing cell polarity. Though not essential for directional sensing or motility, dynacortin is required to establish cell polarity, the third core feature of chemotaxis.

## Background

Dynamic rearrangements of the actin cytoskeleton are required for cell migration, cell polarization, phagocytosis, adhesion, and cytokinesis [1]. This reorganization involves F-actin assembly from soluble monomers in the cytoplasm and their subsequent turnover through depolymerization to replenish the precursor pool [2]. Cells use the force generated from new actin assembly to deform the cell membrane, changing the cell shape to extend the leading edge of the cell. Polymerization of new actin filaments requires actin nucleating factors – Arp2/3 complex and formins – that catalyze new actin assembly, and thus play a key role in inducing morphological changes [3-7]. However, maintenance of the appropriate shape of the cell likely depends on actin cross-linkers to provide mechanical resistance so that focused force production occurs in the right direction.

Dynacortin, an actin filament cross-linking protein, was discovered in *Dictyostelium discoideum *in a genetic screen for suppressors of the cytokinesis defect of *cortexillin-I *mutants [8]. Dynacortin localizes to the cortex and is especially enriched in dynamic protrusions built by the actin cytoskeleton, such as pseudopodia, lamellipodia, and phagocytic cups [8,9]. From a variety of genetic, *in vivo *and *in vitro *analyses, dynacortin has been found to be an actin cross-linking protein that generates mechanical resistance in the cortex that controls cytokinesis contractility dynamics [8-11].

Because of dynacortin's localization to cell surface protrusions in vegetative cells, we speculated that it might play a role in chemotaxis. Here, we use epifluorescence and total internal reflection fluorescence imaging to demonstrate that dynacortin is localized to the actin network, including the leading edges of chemotaxing *Dictyostelium*. Cells depleted of dynacortin can sense chemoattractant but have trouble polarizing normally. Using purified proteins, we demonstrate that dynacortin directly stabilizes actin *in vitro*. Overall, dynacortin is an actin cross-linking protein that facilitates cell polarization during chemotaxis.

## Results

### Dynacortin localization in chemotaxing *Dictyostelium*

**Figure 1:**
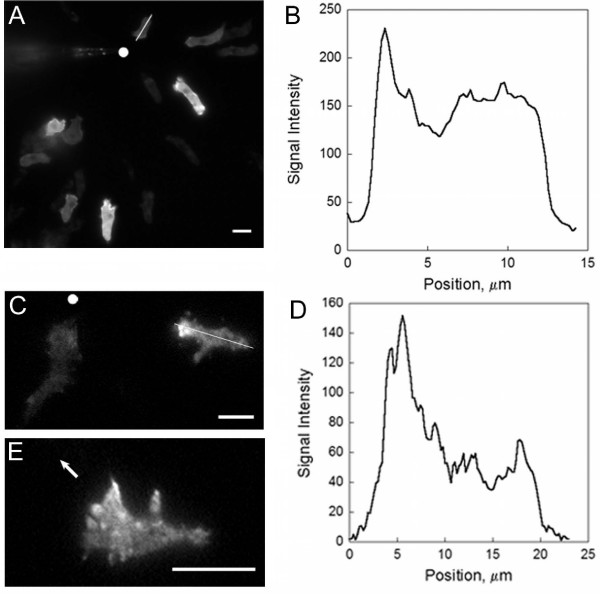
**Dynacortin distribution during cAMP stimulation**. A. GFP-dynacortin localization in wild-type cells was visualized by fluorescence microscopy. The white dot denotes the position of the micropipette. B. Linescan of dynacortin signal intensity of the cell in A marked with a white line. The linescan begins at the front (towards the micropipette) of the cell. C. TIRF imaging of wt: GFP-dyn cells shows dynacortin enrichment in the front half of the cell. The white dot denotes the position of the pipette. D. Linescan of cell in C with the white line. E. High magnification TIRF image of a wt: GFP-dyn cell. The arrow identifies the direction of the micropipette. Dynacortin is localized to cell surface structures that are enriched at the front half of the cell. Scale bars, 10 *μ*m.

In vegetative cells, dynacortin localizes to the cell cortex and is especially enriched in protrusions such as pseudopodia, filopodia, lamellipodia, macropinocytic crowns, and cell-substrate structures called feet or eupodia. When cells change direction, green fluorescent protein fused with dynacortin (GFP-dynacortin) redistributes to the leading edge of the cell [8,9]. This dynamic redistribution of GFP-dynacortin led us to speculate that dynacortin may have a role in chemotaxis. To determine the subcellular localization of dynacortin during chemotaxis, we expressed GFP-dynacortin in wild-type cells. We then imaged GFP-dynacortin distribution during the movement of aggregation-competent cells toward cAMP released from a micropipette. Epifluorescence microscopy revealed that GFP-dynacortin was diffusely localized throughout the cortex with occasional increases in concentration at the leading edge (Figure 1A; Additional file 1). The linescan in Figure 1B demonstrates dynacortin enrichment at the front of the migrating cell.

To obtain higher resolution images, we examined the distribution of dynacortin in the cell surface using total internal reflection fluorescence (TIRF) microscopy (Figure 1C,E; Additional files 2, 3). TIRF imaging allows the cortical layer to be imaged, greatly reducing the signal contribution from soluble GFP-dynacortin in the cytosol (Figure 1D). Dynacortin is organized into fibrous, punctuate structures, which are the actin-rich network near the cell surface that make up the actin feet [9,12,13]. Thus, the actin cross-linker dynacortin is recruited to highly dynamic regions of the cytoskeleton during chemotaxis.

### Dynacortin is required for cell polarization in response to cAMP

**Figure 2:**
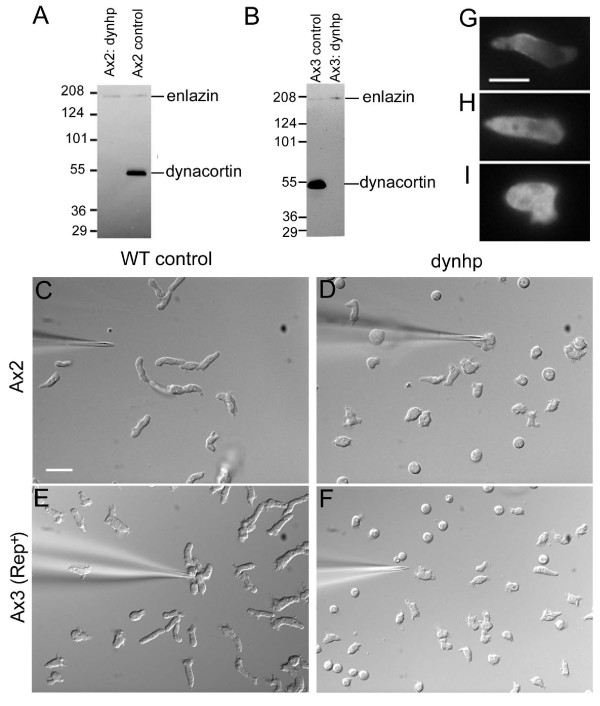
**Dynacortin promotes cell polarization in response to cAMP**. A, B. Western analysis shows that dynacortin is depleted to undetectable levels in an Ax2 and Ax3(Rep orf+) strains. Enlazin [42] is shown as a loading control. C, E. Wild-type control (carrying the empty vector) cells become highly polarized and migrate towards the needle, injecting 1 *μ*M cAMP. D, F. Removal of dynacortin inhibits the ability of the cells to become highly polarized. The fraction of cells that do become polarized move towards the needle with wild-type kinetics (Table 1). Videos are representative of 4 to greater than 12 videos per genotype. Scale bar in C, 20 *μ*m, applies to panels C-F. G. GFP-myoII localized at the rear cortex of the chemotaxing wild-type (*myoII*::GFP-myoII:pDRH; empty vector control) cell. H. In a polarized *dynacortin*-deficient cell (*myoII*::GFP-myoII:pDRH; dynhp:pLD1A15SN), GFP-myoII was partially enriched in the rear half of the cell. I. In a non-polarized *dynacortin*-deficient cell (*myoII*::GFP-myoII:pDRH; dynhp:pLD1A15SN), GFP-myoII did not enrich in the rear of the cell. G-I. Cells are migrating to the right of the panels. Scale bar in G is 10 *μ*m and applies to G-I.

Given the localization of dynacortin during chemotaxis, we tested the chemotaxis ability of cells depleted of dynacortin. Wild-type control cells were able to chemotax with high efficiency and assumed a highly polarized shape in response to cAMP (Figure 2; Additional files 4, 5). Here, we define cell polarization based on cell morphology in which the cells become highly elongated (pilate ellipsoid), and we use quantitative metrics (roundness) to assess cell shape (below). Cells that expressed a *dynacortin *hairpin plasmid had no detectable dynacortin and had a quantitative defect in cell polarization in response to cAMP (Figure 2; Table 1; Additional files 6, 7) [10]. Though we frequently observed that *dynacortin*-deficient cells had highly impaired motility, failing to become polarized (for quantification see Table 1), the degree of immobility was highly variable and wild-type cells also had some highly rounded cells on occasion. Thus, we focused our quantitative analysis on motile cells from wild-type and *dynacortin*-deficient cells and measured the velocity, chemotactic index, directional persistence of the movement and the roundness (Table 1; see Methods for definitions of the parameters). Of the motile cells, *dynacortin*-depleted cells had statistically indistinguishable velocity, chemotactic index and directional persistence as compared to wild-type cells (Table 1). However, *dynacortin*-deficient cells (roundness coefficient: 0.6; Equation 1; Table 1) were significantly more round than wild-type cells (roundness coefficient: 0.4; Table 1). Thus, dynacortin is required for the cell to fully polarize in response to cAMP. We observed similar trends at 5.5 and 7 h of starvation, indicating that the defect in polarization is independent of developmental stage; significant increase in polarization is observed during this time period in wild-type cells [14,15]. Further, even though the *dynacortin*-deficient cells did not polarize as fully as wild-type control cells, GFP-myosin-II partially enriched in the rear half of the polarized, but not non-polarized, *dynacortin*-deficient cells (Figure 2G–I).

**Table 1:** Quantification of dynacortin-deficient chemotaxis

	Fraction of motile cells*		Parameters of motile cells				
Strain	%	n	Velocity (*μ*m/min)	Cos *θ*	Persistence	Roundness†	n

Ax2 control	91%	66	8.2 ± 0.76	0.64 ± 0.040	0.78 ± 0.028	0.44 ± 0.018	31
Ax2: dynhp	31%	109	6.1 ± 1.3	0.57 ± 0.091	0.78 ± 0.064	0.60 ± 0.039	10
Ax3 control	71%	118	4.9 ± 0.26	0.58 ± 0.043	0.74 ± 0.027	0.49 ± 0.014	33
Ax3: dynhp	14%	221	5.0 ± 0.58	0.51 ± 0.11	0.69 ± 0.045	0.62 ± 0.029	10

*Differences in fraction of motile cells between control and dynhp cells for each genetic background were significant (chi-square; p < 0.001).

†Differences in roundness between control and dynhp cells for each genetic background were significant (Student t test; p < 0.001).

All values are mean ± SEM.

**Figure 3:**
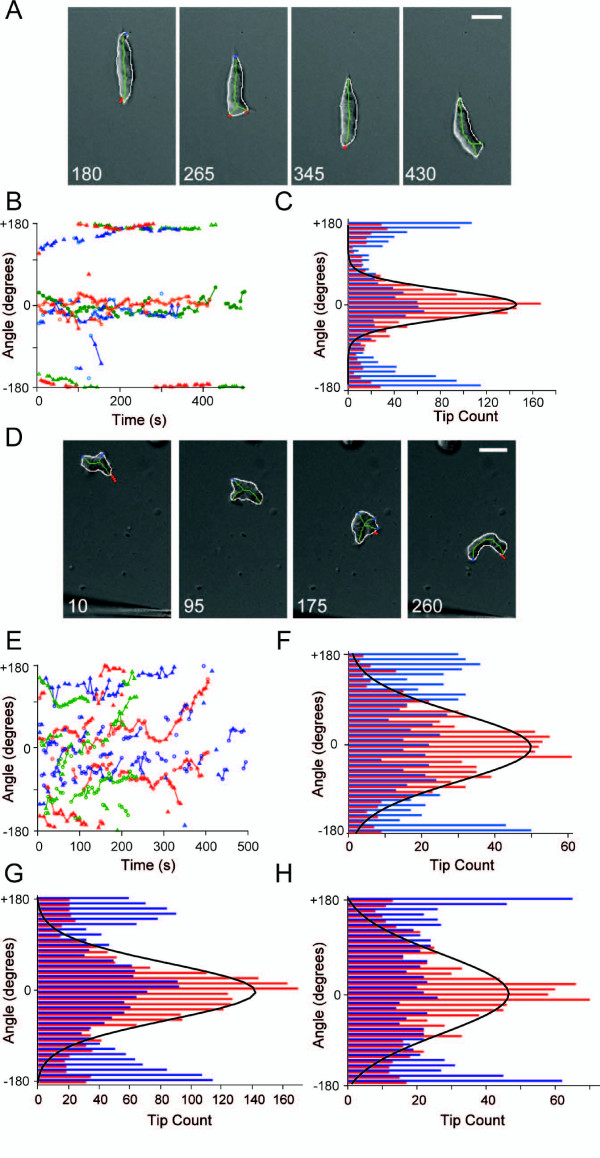
**Without dynacortin, chemotaxing cells send out pseudopods in more diverse directions**. A. Four snapshots from an Ax2 control cell moving towards a needle. The images come from the enclosed video (though the images are rotated to make it easier to fit). They show the cell outline (white), the skeleton (green) and the tips (extensions in red, retractions in blue) in each image. B. The tip angles (relative to the gradient) as a function of time for three representative Ax2 control cells (blue, green and red denote individual cells). Extending and retracting tips are marked with circles and triangles, respectively. Note that leading tips cluster near the 0° angle. C. The extending (red bars) and retracting (blue bars) tip angle distribution for all Ax2 control cells considered (27 cells, 1 606 frames). The black line is a Gaussian curve fit of the extending tips. The mean is at 1.2°, with standard deviation 30.6°. D. Four frames from a video of a chemotaxing Ax2:dynhp cell. E. The tip angles (relative to the gradient) as a function of time for three Ax2:dynhp cells. F. The tip angle distribution (as in C) for all Ax2:dynhp cells (10 cells, 921 frames). The Gaussian fit (black line) yields a mean ± SD of -8.0° ± 65.9° at the front relative to the chemoattractant gradient. G,H. The tip angle distributions for Ax3 control (G) (-2.7° ± 52.8°, n = 29 cells, 2 455 frames) and Ax3:dynhp (H) (-5.5° ± 74.6°, n = 13 cells, 1 100 frames).

To quantify the motility defect further, we used a skeleton representation (see Methods) to characterize the dynamic changes in cellular morphology during chemotaxis (Figure 3). The skeletons of Ax2 control cells consisted primarily of a single segment indicating a lack of lateral protrusions (Figure 3A, Additional file 8). With the skeleton representation, we could separate the extension (red tips) and retraction (blue tips) of cell surface protrusions. The extending tips were relatively long-lived (Figure 3B) and the angles (mean ± SD: 1.2° ± 30.6°; Equation 2) of the extending tips co-aligned with the chemoattractant gradient (Figure 3C). In contrast, the skeletons of Ax2:dynhp cells showed multiple branches characteristic of cells with multiple protrusions (Figure 3D, Additional file 9). These projections occurred at a broader range of angles relative to the gradient and had a shorter duration (Figure 3E). Moreover, though these projections were also in alignment with the chemoattractant gradient on average, a broader distribution of extending protrusion angles was observed (mean ± SD: -8.0° ± 65.9°) (Figure 3F). Similarly, Ax3 control (mean ± SD: -2.7° ± 52.8°) and Ax3:dynhp cells (mean ± SD: -5.5° ± 74.6°) also produced protrusions with a similar angle relative to the gradient but Ax3:dynhp cells extended the protrusions with a larger variation in angles (Figure 3G,H). Thus, without dynacortin, cells generate more protrusions over a much broader distribution of angles than wild-type cells do.

**Figure 4:**
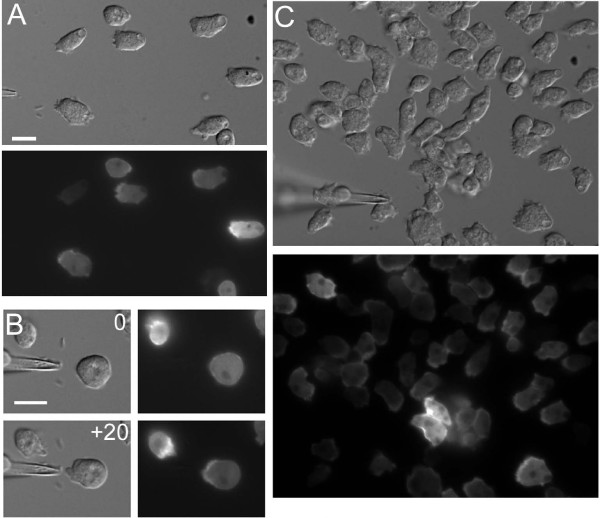
**GFP-dynacortin localizes to the front end of cells in the presence of LY29004, a PI3 kinase inhibitor**. A. Prior to LY29004 treatment, GFP-dynacortin was enriched at the leading edge (towards the needle) of the cell. B. Within 30 s of LY29004-treatment, the cells became rounded and GFP-dynacortin was uniformly distributed (0). While in 50-*μ*M LY29004, the cell extended a pseudopod enriched in GFP-dynacortin towards the needle 20 s after bringing the needle up to the cell (+20). C. Within 3 min of LY29004-treatment, the LY29004 cells resumed their ability to move towards the needle, but without any clear polarization. GFP-dynacortin still enriched at the front of these cells. Scale bars, 10 *μ*m. Bar in A also applies to C.

The defect in polarization of dynacortin-deficient cells is similar to that observed for LY29004 (an inhibitor of PI3 kinase activity) treatment [14,15]. Therefore, we investigated the effect of LY29004 treatment of GFP-dynacortin localization (Figure 4). Using 5.5-h cells, wild-type cells expressing GFP-dynacortin were elongated with GFP-dynacortin enriched at the leading edge of the cell (Figure 4A). Within about 30 s of treating the cells with 50-*μ*M LY29004, the cells nearly completely rounded up as described previously (Figure 4B) [14]. However, by bringing the needle containing 1 *μ*M cAMP close to the rounded cell, the cell accumulated GFP-dynacortin on the side facing the needle and extended a pseudopod within 20 s (Figure 4B). Within 2–3 min, the cells adapted to the LY29004 and began to chemotax towards the needle but never fully repolarized (Figure 4C). Significantly, GFP-dynacortin accumulated at the front end of the chemotacting LY29004-treated cells, suggesting that dynacortin accumulation at the cell front may be independent of PI3 kinase activity. We also treated dynacortin-deficient cells with LY29004; however, while the cells again showed lower overall polarization relative to wild-type cells, they were still able to chemotax towards the needle (data not shown).

For a population assessment, we examined the development on DB-agar. The *dynacortin*-depleted cells appeared largely indistinguishable from the wild-type control cells, forming normal looking fruiting bodies. However, at decreasing densities, the wild-type control cells repeatedly formed large, extensive streams whereas the *dynacortin*-depleted cells seldom formed streams, and when they did they were smaller than the wild-type control. Thus, in this population assay, dynacortin is required for development at low cell densities, consistent with the overall polarization and motility defects observed in the needle assays.

### Dynacortin does not affect bulk actin polymerization in response to cAMP stimulation

**Figure 5:**
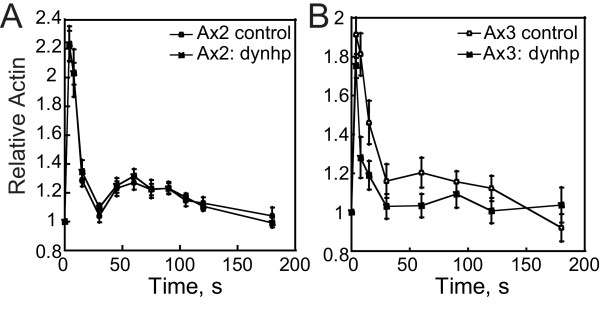
**Dynacortin is not required for bulk actin assembly in response to cAMP**. A. Comparison of F-actin polymerization responses in wt control cells (Ax2) carrying control plasmid (pLD1) or an expression plasmid for dynacortin hairpin (wt: dynhp). wt control (n = 12); wt: dynhp (n = 12). B. Comparison of F-actin polymerization responses in wild-type (Ax3(Rep orf+)) control cells carrying control plasmid (pLD1) or an expression plasmid for dynacortin hairpin (wt: dynhp). wt control (n = 8); wt: dynhp (n = 5).

As dynacortin localizes to actin-rich dynamic cortical domains, we determined whether dynacortin contributes to the bulk actin polymerization dynamics in the cell. We compared the kinetics of *in vivo *bulk actin polymerization of *dynacortin*-silenced cells (wt: *dynhp*) to control wild-type cells in response to a chemotactic stimulus (Figure 5). Within 6 s of stimulation with 1-*μ*M cAMP, wild-type and *dynacortin*-depleted cells showed a transient assembly of actin (~two fold), and then a decrease to basal levels by 30 s, followed by a smaller second (~1.2-fold) peak at 90 s. Thus, dynacortin does not have a detectable effect on the bulk actin assembly dynamics in response to cAMP. Further, basal actin levels were similar between wild-type and *dynacortin*-depleted cells (data not shown).

### Dynacortin drives polymerization under low-ionic strength conditions

Because dynacortin enriches at the leading edge of chemotaxing cells and these motile cells are known to assemble new filamentous actin when stimulated with cAMP, we wondered whether dynacortin has an effect on actin assembly. To do this, we investigated the effect of dynacortin on actin assembly *in vitro *using pyrene-actin assays. In these experiments, the fluorescence of pyrene-actin increases as it assembles into filaments. Traditionally, these assays are performed in high-salt F-buffer, which contains 50 mM KCl and 1 mM MgCl_2_. However, while performing some control experiments, we discovered that dynacortin drove actin assembly in G-buffer, which contains only 10 mM Tris-HCl and 0.2 mM CaCl_2_. Because G-actin normally does not assemble under these low-salt G-buffer conditions, this dynacortin-mediated assembly represents a nearly infinite rate enhancement. Therefore, we tested dynacortin's role in actin assembly under both G- and F-buffer conditions and report these findings here.

We first measured the critical concentration of G-actin required for polymerization in the presence and absence of dynacortin. The critical concentration of G-actin assembled by 2.5 *μ*M dynacortin under G-buffer conditions was only 0.03 *μ*M, which is significantly lower than the 0.17 *μ*M critical concentration for actin alone assembled by the addition of F-buffer (Table 2). Because the addition of dynacortin to F-actin caused a partial quenching of pyrene fluorescence, the critical concentration of actin under F-salt conditions could not be determined accurately (Table 2).

**Table 2:** Critical concentrations of dynacortin-mediated actin assembly

Protein	Critical concentration Mean ± SEM (n)
Actin:	
F-buffer	0.17 ± 0.017 *μ*M (3)
F-buffer + Dyn_2_	Not measurable
G-buffer	No assembly
G-buffer + 2.5 *μ*M Dyn_2_	0.03 ± 0.016 *μ*M (3)

Dynacortin_2_:	
G-buffer + 5–10 *μ*M Actin	1.7 ± 0.8 *μ*M (8)

**Figure 6:**
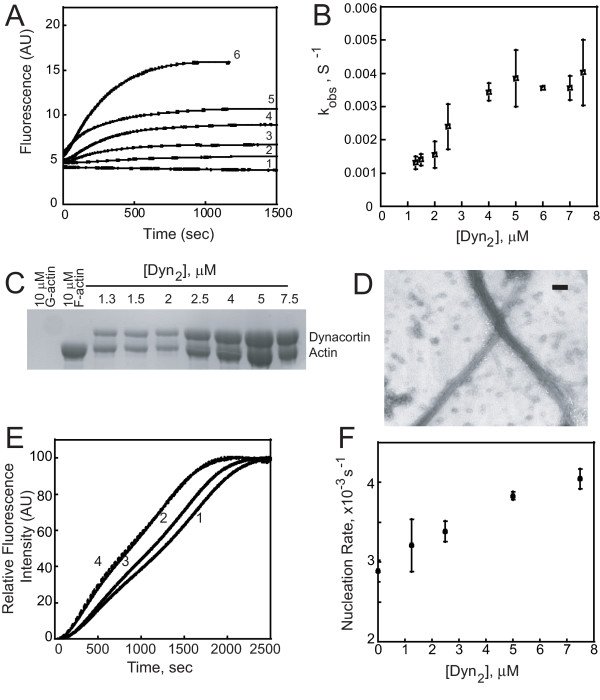
**Dynacortin promotes assembly of actin in low-salt conditions *in vitro***. A. Actin (10 *μ*M) was induced to polymerize under G-buffer conditions. Polymerization was monitored as an increase in fluorescence intensity over time by exciting at 365 nm and collecting emission at 385 nm. The experiment was repeated at least three times at each dynacortin concentration with the same results. Curve 1, 10 *μ*M G-actin alone; curve 2, 10 *μ*M G-actin + 1.3 *μ*M Dyn_2_; curve 3, + 2.5 *μ*M Dyn_2_; curve 4, + 4 *μ*M Dyn_2_; curve 5, + 5 *μ*M Dyn_2_; curve 6, + 7.5 *μ*M Dyn_2_. B. Observed rates for actin assembly induced by varying concentrations of dynacortin. The slope of the polymerization curve was determined by fitting polymerization data to Equation 3. The slope of the line-fitting the rising part of the curve gives a value of *k*^+^/*K*_*M *_= 0.0014 *μ*M^-1^s^-1 ^and slope of the line-fitting the plateau part of the curve yields a value of *k*^+ ^= 0.0042 ± 0.0009 s^-1^. The calculated *K*_*M *_is 3 *μ*M. C. Dynacortin binds to G-actin. Varying concentrations of Dyn_2 _and 10-*μ*M skeletal muscle actin were mixed in G-buffer, ultracentrifuged and the pellets were analyzed by SDS-PAGE. Concentrations are indicated on gel. D. Dynacortin induced the formation of actin bundles under G-buffer condition. Electron micrograph of grid spotted with polymerization reaction mixture of 3-*μ*M pyrene-labeled actin and 3-*μ*M Dyn_2 _1 000 s after assembly was initiated. Scale bar, 200 nm. E. Dynacortin decreased slightly the lag time of actin assembly under F-buffer conditions. A total of 2.5 *μ*M actin was present in each sample. Curve 1, 2.5 *μ*M actin alone; curve 2, 1.3 *μ*M Dyn_2_; curve 3, 5 *μ*M Dyn_2_; curve 4, 7.5 *μ*M Dyn_2_. F. Dynacortin increased the apparent nucleation rate nearly twofold in a concentration-dependent manner.

Then, we investigated the impact of dynacortin on the kinetics of actin polymerization under G-buffer conditions. We mixed increasing amounts of dynacortin with a constant amount of pyrene-labeled Ca^2+^-actin (10 *μ*M) and the rate of polymerization was monitored over time by fluorescence intensity measurements. At dynacortin concentrations above 2 *μ*M (actin:dynacortin ratio of 5:1), the G-actin assembled into polymers, and dynacortin increased the steady state amount of assembled actin in a dynacortin-concentration-dependent manner (Figure 6A,B). Because of the absence of an apparent lag phase (Figure 6A), the observed rate of polymerization could be obtained by fitting the curves to an exponential rise equation (Equation 3). The rate of polymerization increased linearly with increasing dynacortin concentration until a maximal value (*k*^+ ^= 0.004 s^-1^) was reached at 5-*μ*M dynacortin. Further increase in dynacortin concentration resulted in no increase in rate beyond the maximal value. Considering the *k*^+ ^and the slope of the concentration-dependent phase, the *K*_*M *_was calculated to be 3 *μ*M, which is similar to the measured 1.7 *μ*M critical concentration for dynacortin to assemble polymers in G-buffer (Table 2). These results suggest that under low ionic strength conditions, dynacortin scaffolds actin monomers into filaments. Further, the absence of an apparent lag phase suggests that the mechanism of dynacortin-mediated actin assembly is different than the conventional nucleation-elongation mechanism of actin assembly.

Next, we verified that the increase in fluorescence was not due to an effect on the fluorescent probe but that dynacortin indeed bound actin under these low-salt conditions, using a co-sedimentation assay (Figure 6C). The solutions from the pyrene assay were centrifuged at high-speed centrifugation to pellet all the assembled actin and bound dynacortin. In the case of G-actin alone, no actin precipitated; most of the actin assembled using F-buffer precipitated at this speed. In the presence of G-actin and dynacortin, dynacortin co-precipitated with actin and had a saturation stoichiometry of one Dyn_2 _dimer per actin monomer. This saturation stoichiometry is identical to the saturation stoichiometry for dynacortin-mediated actin bundling [9,10]. More importantly, the amount of actin that assembled and pelleted increased as the dynacortin concentration increased. To independently confirm the presence of actin filaments, we examined the samples under the electron microscope and observed bundles of actin filaments in the presence of dynacortin under low salt conditions (Figure 6D). Staining actin filaments with rhodamine-phalloidin and visualizing them under the fluorescence microscope also revealed the presence of bundles (data not shown). Taken together, these data demonstrate that dynacortin drives assembly of G-actin into polymers under conditions that disfavor assembly and that the amount of polymer formed is proportional to the amount of dynacortin available. Thus, dynacortin acts as a scaffold or molecular staple that holds the actin monomers together under these low salt G-buffer conditions.

We also tested whether dynacortin can nucleate actin under standard F-buffer conditions. Under these conditions, the conventional lag phase associated with the nucleation-elongation reaction is observable. As the dynacortin concentration was increased, the lag phase for actin assembly decreased slightly (Figure 6E). The lag phase is derived from the time required for actin nuclei to form. To analyze this more quantitatively, we determined the time for the actin fluorescence signal to increase to 10% of the maximum signal, indicating that the actin is 10% assembled (Equation 4). The reciprocal of the time to 10% has been demonstrated to be a close approximation of the nucleation rate [16]. Dynacortin weakly increased the apparent nucleation rate in a dynacortin-concentration-dependent manner (Figure 6F). Overall, we conclude that dynacortin's primary role is in actin filament cross-linking with a possible effect on filament stability.

## Discussion

Chemotaxis is an important cellular process for organisms, ranging from bacteria and protists to higher multicellular animals [17]. In mammals, it is critical for normal immune function, while in the simple protozoan *Dictyostelium discoideum*, it is important for coalescence of individual cells into multicellular structures that allow the organism to respond to environmental stresses such as nutrient starvation. Chemotaxis can be separated into three components: directional sensing, polarization and motility [18]. Although a high level of understanding has been achieved for directional sensing and motility, little is known about how cells polarize and how these three processes are integrated is not at all understood. Certainly, evidence exists that the actin network reinforces directional sensing [19,20]. Because of the complexity of actin assembly, motility, and signaling, it is possible that cooperativity and redundancy have obscured critical players in genetic screens geared to uncover the connections between sensing and motility. Also, the usual suspects for motility (Arp2/3, Scar, and Wasp) play a role in the chemotactic response [21-23]. Our results demonstrate that the actin cross-linking protein, dynacortin, plays a direct role in cell polarization during chemotaxis.

Dynacortin is a novel actin cross-linking protein that enriches in cell surface protrusions as well as punctate surface structures that are the *Dictyostelium *equivalents of focal adhesions [8,9]. Dynacortin contributes to cell cortex mechanics and interacts antagonistically with myosin-II driven dynamic rearrangements of the cell cortex [9-11,24]. By enriching at sites of dynamic actin cytoskeleton, dynacortin-mediated cross-linking and polymer stabilization may create the appropriate level of viscoelastic resistance for the cell to polarize and/or maintain a polarized shape. Cortical viscoelasticity may also contribute to chemotaxis by setting a timescale over which a cell can change shape as it sends out a pseudopod. A cell with greater viscoelasticity would be expected to be slower at assembling and disassembling a pseudopod whereas a cell with lower viscoelasticity should be able to form pseudopods much more rapidly. Indeed, the softer dynacortin cells form many more pseudopods per unit of time and extend them in a greater number of directions. Different levels of mechanical resistance between the leading and trailing edges of chemotactic cells have been documented previously, and the membrane-actin tether, talin, contributes significantly to the cortical resistance at the rear of the cell [25].

Different regulatory systems modulate the actin responses during chemotaxis. In mammalian cells, the initial actin response is thought to be mediated by cofilin [26]. However, in *Dictyostelium*, actin assembly appears to be regulated at least in part by PTEN phosphatase and the PI3 kinases. Inhibition of these phosphoinositol-regulating enzymes may alter the general cytoskeletal dynamics, leading to defects in chemotaxis [15,27]. Dynacortin, in contrast, may act downstream of the signaling networks to directly modulate the viscoelasticity of the actin network thereby promoting polarization in response to chemotactic stimulation.

In addition to cross-linking, our *in vitro *data suggest that dynacortin may serve as a scaffolder of actin assembly. Under low-salt conditions, dynacortin drove monomeric actin into polymers in a concentration-dependent manner. Further, the amount of assembled polymer was dependent on the dynacortin amounts and assembly occurred with a 1:1 stoichiometry, indicating that dynacortin scaffolded the actin. Other examples of actin scaffolding proteins include nebulin, which controls the length of the actin filaments in the muscle sarcomere [28,29], and the *Salmonella *sipA, which promotes cytoskeletal reorganization for entry into the cell [30-32]. Thus, while this scaffolding activity is unusual, it is not unprecedented.

## Conclusion

Regulated actin assembly is the essential component for directed cell motility. Because of its importance for normal cell physiology, numerous players contribute to the assembly and organization of the leading edge actin network. While Arp2/3 and its regulators have critical roles, other accessory proteins such as actin cross-linkers also contribute, ensuring robustness. Our results demonstrate that the actin cross-linker dynacortin should be considered in chemotaxis for its role in cross-linking and stabilizing the dynamic actin network, promoting cell polarization. Indeed, the cytoskeleton is the prototypical example of biocomplexity where many proteins that are physically connected by cytoskeletal filaments interact to promote complicated cell behavior.

## Methods

### Cell culture, development and chemotaxis assay

*Dictyostelium discoideum *Ax3(Rep orf+) [8], Ax2, or *myoII*::GFPmyoII:pDRH, HygR, RFP-*α*-tubulin:pDxA-Bl cells were cultivated in Hans' enriched HL-5 medium in polystyrene Petri dishes at 22°C [8]. Wild-type (wt) control and wt: dynhp cells were constructed as previously described [10]. Control cells contain the empty vector, pLD1A15SN. Western analysis using anti-dynacortin antibodies was used to detect dynacortin levels in control and wt: dynhp cells [8,10].

For development, cells were washed in developmental buffer (DB; 10 mM phosphate buffer, 2 mM MgSO_4_, 0.2 mM CaCl_2_) and plated on DB agar at known densities (cells/cm^2^). Twofold dilution series from 1 × 10^6 ^to 6 × 10^4 ^cells/cm^2 ^were performed three times in duplicate (total n = 6 for each genotype). Development was monitored for up to 30 h.

To starve cells for chemotaxis assays, cells were washed in DB buffer, resuspended at 2 × 10^7 ^cells/mL in 5 ml DB and rotated at 100 rpm for 1 h. Cells were then pulsed with 50-nM cAMP at 6 min intervals for 4–6.5 h. For chemotaxis assays, 1-*μ*M cAMP was released from a microcapillary pipette (0.5 *μ*m inner diameter) with 40 hPa of pressure. Cells were imaged using an Olympus IX81 motorized microscope using a 40 × (numerical aperture (NA) 1.3) objective and a 1.0× optivar. Images were collected every 5 s for 20 min using Metamorph imaging software (Molecular Devices Corp., Downingtown, PA USA).

To quantify chemotaxis, we first determined the fraction of cells that were motile. To do this, we followed all of the cells that were present in the initial field of view; this eliminates the impact of cells that migrate into the field, which would lead to an overestimation of the percentage of motile cells. To analyze the motile cells, we determined the velocity [33], chemotactic index (cosΘ) [27], directional persistence [34], and roundness [33]. The fraction of chemotactic cells was determined by following the behavior of every cell in the initial microscopic field over a 20 min video recording. For the detailed analysis of motile cells, we only followed cells that did not collide with another cell during the movie to avoid complications from obstacles. Analysis was performed using a Matlab Image Processing Toolbox (Mathworks, Natick, MA, USA). Individual cells were first segmented and tracked during the course of each video. The centroid was then monitored at 15-s time resolution for the duration of the video. Similarly, we analyzed cell motility by monitoring the centroid position of each cell at 2-min time resolution using Image J [35]. The automated (Matlab) and manual (Image J) methods gave statistically identical results. Velocity reported was the mean velocity of each cell as measured using 15-s time windows. Directional persistence is the net path taken by the cell over the entire video divided by the total path taken using a 15-s time resolution. The chemotactic index (cosΘ) is the cosine of the angle defined by the cell position at time t_+15s _to the cell position at time t and from the cell position at time t to the needle; thus, the initial position of the cell at time t is the vertex of the angle. Roundness (R) is calculated as:

R = 4*π*A/L^2^

Where A is the area of the cell and L is the perimeter length. By this calculation, a circular cell will have an R-value of 1 and a line segment will have an R-value of 0. Each cell had a mean velocity, cosΘ, directional persistence, and roundness, which were averaged for all cells within the genotype. Standard errors of the mean were calculated from the distribution of means for each genotype. Values are presented as mean ± standard error of the mean (SEM). Student t tests were used to evaluate statistical significance of differences between control and *dynacortin*-depleted strains for the various parameters.

### Quantification of pseudopod formation dynamics using skeletons

Image analysis of pseudopod formation dynamics was based on a skeleton representation of cell shape [36] and the detection of shape changes between successive frames, using the Matlab Image Processing Toolbox (Mathworks). First, cells were segmented and tracked. For each frame, the boundary of the segmented image was smoothed by curve fitting using recursive least-square estimation [37]. For each frame, the skeleton of each shape was computed. These were pruned in two steps. To avoid spurious branches, a length threshold (8 pixels of length – approximately 2.46 *μ*m) was set in the first step. Single branches whose length did not exceed the threshold were eliminated; multiple branches with comparable lengths that were shorter than the threshold were combined from their common root by averaging their terminal locations. The second pruning step takes into account the changes over time of the cell shape. To this end, cell shape differences between the current and previous frame were detected. Skeleton tips were then tested to see whether they were close to the areas with cell shape differences. Those farther than 1.8 *μ*m were eliminated. Also deleted were the tips that did not point towards areas in which the cell shape changed. Finally, branches were classified as extensions or retractions depending on whether they pointed towards regions in which the cell shape was expanding or shrinking. Tip angles are defined as the angle between the rays emanating from the cell's centroid to the needle location and extension/retraction tips. To determine the time course of the tips, differences in tip angle between up to four successive frames were computed and compared to an arbitrary threshold of 60°.

Angle distributions for the extending tips were fitted by a Gaussian with finite support:

cσ2πe−(θ−μ)2/2σ2,θ∈[−180∘,180∘)
 MathType@MTEF@5@5@+=feaafiart1ev1aaatCvAUfKttLearuWrP9MDH5MBPbIqV92AaeXatLxBI9gBaebbnrfifHhDYfgasaacPC6xNi=xI8qiVKYPFjYdHaVhbbf9v8qqaqFr0xc9vqFj0dXdbba91qpepeI8k8fiI+fsY=rqGqVepae9pg0db9vqaiVgFr0xfr=xfr=xc9adbaqaaeGacaGaaiaabeqaaeqabiWaaaGcbaqbaeqabeGaaaqaaKqbaoaalaaabaGaem4yamgabaacciGae83Wdm3aaOaaaeaacqaIYaGmcqWFapaCaeqaaaaakiabdwgaLnaaCaaaleqabaGaeyOeI0IaeiikaGIae8hUdeNaeyOeI0Iae8hVd0MaeiykaKYaaWbaaWqabeaacqaIYaGmaaWccqGGVaWlcqaIYaGmcqWFdpWCdaahaaadbeqaaiabikdaYaaaaaGccqGGSaalaeaacqWF4oqCcqGHiiIZcqGGBbWwcqGHsislcqaIXaqmcqaI4aaocqaIWaamdaahaaWcbeqaaiablIHiVbaakiabcYcaSiabigdaXiabiIda4iabicdaWmaaCaaaleqabaGaeSigI8gaaOGaeiykaKcaaaaa@5196@

The parameters *μ *and *σ *were obtained by optimization using the simplex search method in Matlab.

### Epifluorescence and total internal reflection fluorescence (TIRF) imaging

Wild-type GPP-dynacortin (wt: GFP-dyn) cells were imaged on an Olympus IX81 epifluorescence/total internal reflection fluorescence (TIRF) microscope. Cells were imaged in DB buffer. For epifluorescence imaging, cells were illuminated with a Xenon lamp, and a 40 ×, 1.3 NA oil-immersion objective with a 1.6× optivar were used for image collection. For TIRF imaging, cells were illuminated with a 488-nm laser, and a 60 × 1.45 NA objective and 1.6× optivar were used for image collection. Images were collected using Metamorph Software (Molecular Devices) and processed using Image J [35] and Adobe Photoshop (Adobe Systems, Inc., San Jose, CA USA). For GFP-myoII imaging, *myoII *(mhcA; HS1 [38]) cells complemented with GFP-myoII: pDRH [39] and expressing either the empty pLD1A15SN (control) vector or pLD1A15SN:dynhp [10] were used.

### Actin concentration determination and in vivo actin polymerization assay

The relative filamentous actin content was determined from tetramethylrhodamine B isothiocyanate-phalloidin (TRITC-phalloidin) staining of *Dictyostelium *cells [27]. Briefly, developed cells were resuspended at 3 × 10^7 ^cells/mL with 3 mM caffeine in PM buffer (10 mM phosphate buffer, 2 mM MgSO_4_) and shaken at 200 rpm for 15–30 min. At various times after 1-*μ*M cAMP addition, 100-*μ*L aliquots were collected, fixed and stained with TRITC-phalloidin. F-actin was proportional to the amount of phalloidin fluorescence (excited at 540 nm and emitted at 570 nm) extracted from cell pellets.

### Purification of actin and dynacortin

Chicken skeletal muscle actin was purified from acetone powder [40], gel filtered and labeled with pyrenyl-maleimide [41]. Recombinant dynacortin was purified following the method described previously [10].

### Actin dynamics by fluorescence spectroscopy

Dynacortin and pyrene-labeled actin (10% and 60% pyrene-labeled) in G-buffer (10 mM Tris-HCl, pH 7.5, 0.2 mM CaCl_2_, 0.2 mM ATP, 5 mM DTT) was precleared at 100 000 *g *for 1 h before use. To assess the effect of dynacortin on actin polymerization, 10-*μ*M pyrene-labeled actin was mixed with varying concentration of dynacortin under G-buffer conditions. No difference was observed in dynacortin-mediated G-actin assembly in G-buffer conditions between 10% and 60% pyrene-labeled actin. Changes in fluorescence were monitored by exciting at 365 nm and collecting emission at 385 nm. The rate of polymerization, monitored by the change in fluorescence signal, was calculated using the relationship:

F_t _= F_max _- F_initial_·e^-*k*t^

Where F_t _is the fluorescence amplitude at any given time t, F_max _is the end point, and F_initial _is the starting point of the fluorescence amplitude, and *k *is the polymerization rate constant determined from fitting the polymerization curve to the above equation.

For nucleation in F-buffer conditions, varying concentrations of dynacortin were added to 2.5 *μ*M G-actin (10% pyrene-labeled) in F-buffer (G-buffer plus 2 mM MgCl_2 _and 50 mM KCl). Nucleation rates were calculated by determining the time required to achieve 10% polymerization (0.1(F_max_-F_initial_)) as described in [16]:

Rate_nucleation _= 1/(t_(0.1(Fmax-Finitial))_).

This is only an approximation of the nucleation rate as nucleation involves multiple steps.

### Cosedimentation assay

The samples from the fluorescence assay were centrifuged for 30 min at 100 000 *g *in a TLA 100 rotor. Only polymerized actin sediments under these conditions. The pellets were resuspended in 1 × Laemmli's sample buffer and equivalent amounts were loaded on 15% SDS-PAGE gels. Densitometry of Coomassie Blue stained gels was used to determine the fraction of actin that was polymerized by dynacortin.

### Critical concentration determination

10-*μ*M G-actin (10% pyrene-labeled) was polymerized by addition of 2 mM MgCl_2 _and 50 mM KCl 25°C for 1 h. F-actin was diluted to the desired concentration in F-buffer. The pyrene fluorescence of F-actin at each concentration was monitored as described above. Unassembled G-actin pyrene fluorescence at each concentration was also measured to determine the background fluorescence. To determine the critical concentration of actin or dynacortin, the net change in pyrene fluorescence of an actin polymerization reaction was measured as a function of increasing actin or dynacortin concentration. Critical concentrations were obtained from the linear fits of the data, which were used to determine the x-intersect of the comparator unassembled and assembled samples.

### Electron microscopy

Samples containing actin and dynacortin were deposited on carbon-coated Formvar grids, negatively stained with 1.5% uranyl acetate, and observed using a Phillips CM-120 TEM, operating at 80 kV.

## Authors' contributions

CK, RM, EMR, and JK performed the cell-based experiments. RM performed all biochemical studies. YX and PAI developed image analysis software, performed quantitative analysis of chemotaxis parameters, and contributed to the writing of the paper. DNR conceived of the project, oversaw the research, and primarily wrote the paper. All authors read, edited, and approved the final manuscript.

## Supplementary Material

Additional file 1 **GFP dynacortin concentrates along the cortex and enriches at the leading edge during chemotaxis**. Video is imaged using wide-field epifluorescence with a 40 × (NA 1.3) objective and a 1.6× optivar. Frames are queued every 15 s. Click here for file

Additional file 2 **GFP dynacortin concentrates along the surface at the leading edge and at sites of cell protrusions during chemotaxis**. Video is imaged using total internal reflection fluorescence microscopy using a 60 × (NA 1.45) 1.6× optivar. Frames are queued every 1 s. Click here for file

Additional file 3 **GFP dynacortin concentrates along the surface at the leading edge and at sites of cell protrusions during chemotaxis**. Video is imaged using total internal reflection fluorescence microscopy using a 60 × (NA 1.45) 1.6× optivar. Frames are queued every 1 s. Click here for file

Additional file 4 **Wild-type (Ax2) control (carrying the empty vector) cells become highly polarized and move smoothly towards a needle injecting 1 *μ*M cAMP**. Video is imaged using differential interference contrast imaging with a 40 × (NA 1.3) objective and a 1× optivar. Frames are queued every 5 s. Click here for file

Additional file 5 **Wild-type (Ax3(Rep orf+)) control (carrying the empty vector) cells become highly polarized and move smoothly towards a needle injecting 1 *μ*M cAMP**. Video is imaged using differential interference contrast imaging with a 40 × (NA 1.3) objective and a 1× optivar. Frames are queued every 5 s. Click here for file

Additional file 6 **Wild-type (Ax2):dynhp cells do not become highly polarized and have trouble moving towards the needle injecting 1 *μ*M cAMP**. Video is imaged using differential interference contrast imaging with a 40 × (NA 1.3) objective and a 1× optivar. Frames are queued every 5 s. Click here for file

Additional file 7 **Wild-type (Ax3(Rep orf+)):dynhp cells do not become highly polarized and have trouble moving towards the needle injecting 1 *μ*M cAMP**. Video is imaged using differential interference contrast imaging with a 40 × (NA 1.3) objective and a 1× optivar. Frames are queued every 5 s. Click here for file

Additional file 8 **Wild-type (Ax2:pLD1) control cell showing the skeleton as it moves towards the needle located to the left of the field. The skeleton is computed and displayed at each frame (green bar)**. Extending pseudopods are marked with red tips and retracting pseudopods are marked with blue tips. Note that the green skeleton represents the shape of the cell in the current frame (frame n). The red tips are generated from the difference between the n and n + 1 frames of the video. Because the red tips mark extending pseudopods, they will not link to the green skeleton in the first frame that they appear but will link in the subsequent frame. The blue tips are also generated from the difference between the n and n + 1 frames but because they are retracting they always link to the green skeleton. Video is imaged using differential interference contrast imaging with a 40 × (NA 1.3) objective and a 1× optivar. Frames are queued every 5 s. Click here for file

Additional file 9 **Wild-type (Ax2): dynhp cell showing the skeleton as it moves towards the needle located to the bottom of the field**. The skeleton is computed and displayed at each frame (green bar). Extending pseudopods are marked with red tips and retracting pseudopods are marked with blue tips. Here the cell sends out multiple pseudopods at a time and at a variety of angles. Note that the green skeleton represents the shape of the cell in the current frame (frame n). The red tips are generated from the difference between the n and n + 1 frames of the video. Because the red tips mark extending pseudopods, they will not link to the green skeleton in the first frame that they appear but will link in the subsequent frame. The blue tips are also generated from the difference between the n and n + 1 frames but because they are retracting they always link to the green skeleton. Video is imaged using differential interference contrast imaging with a 40 × (NA 1.3) objective and a 1× optivar. Frames are queued every 5 s. Click here for file
